# Contribution of Morphology of Frontal Sinuses (Linear and Volumetric Measurements) to Gender Identification Based on Cone Beam Computed Tomography Images (CBCT): A Systematic Review

**DOI:** 10.3390/jpm13030480

**Published:** 2023-03-07

**Authors:** Anastasia Mitsea, Nikolaos Christoloukas, Aliki Rontogianni, Christos Angelopoulos

**Affiliations:** 1 Department of Oral Diagnosis and Radiology, School of Dentistry, National and Kapodistrian University of Athens, 11527 Athens, Greece; 2 Department of Orthodontics, School of Dentistry, National and Kapodistrian University of Athens, 11527 Athens, Greece

**Keywords:** frontal sinus, cone beam computed tomography, forensic identification, gender

## Abstract

Human identification is considered as an important step in the reconstruction of biological profiles, especially of unknown individuals. Frontal sinuses (FS) have been regarded as an ideal anatomical structure for individualisation because few pathological conditions can potentially alter their shape. Aim: The aim of this review was to evaluate scientific evidence published since January 2010 and determine whether the dimensions and volume of FS might be useful parameters for gender determination and human identification, based only on cone beam computed tomography images (CBCT). Methods: This review was performed in accordance with the PRISMA statement. Four databases were searched for articles published between January 2010 and June 2022. Results: A total of 195 records were initially identified, and 90 remained after a manual duplicate check. Eight articles were selected for a full review according to the inclusion and exclusion criteria after title and abstract screening. A total of 718 participants (359 males and 359 females) were identified from the included studies. Frontal sinus volume (FSV) was significantly higher in male individuals. Frontal sinus height (FSH) and volume were the superior discriminating parameters for forensic identification. Conclusions: This review demonstrates that assessment of FS based on CBCT images could be beneficial for gender identification in forensic science. According to the obtained studies, frontal sinus volume (FSV) and frontal sinus height (FSH) are significant greater in males than in females, providing an additional complementary method. Larger sample size and common measurement protocols are needed to verify their usefulness.

## 1. Introduction

Human identification is considered as an important step in the reconstruction of biological profiles, especially in unknown individuals. This identification is more reliable in adults [1]. Human skeletons’ bones have occasionally been used for gender determination (pelvis, skull, long bones), which are usually fractured or incomplete (due to explosions, natural disasters, etc.) [2]. In the forensic sciences, radiology plays an essential role as a non-invasive and objective examination approach. The contribution of radiology is useful in several sectors of forensic investigation, particularly in the identification of unidentified human remains [3]. Details of bones, such as volume, size, shape, and individual characteristics, constitute consistent evidence. When radiographed, this evidence can be utilised to identify unidentified corpses or remains [4].

Gender dimorphism is apparent in several anatomical features of the skull [5]. Quite often in forensic cases, only a portion of the skull may be available. Consequently, it is advantageous to be able to detect gender-specific traits of the skull. Among the gender-specific traits of this skeleton component are the foramen magnum diameters and bicondylar width. Moreover, according to recent research data, the condylar height, the coronoid height, and the superior border of the mental foramen and ramus are substantially higher in male individuals. Assessing numerous parameters simultaneously enhances the accuracy of gender estimation [5,6,7].

**Figure 1:**
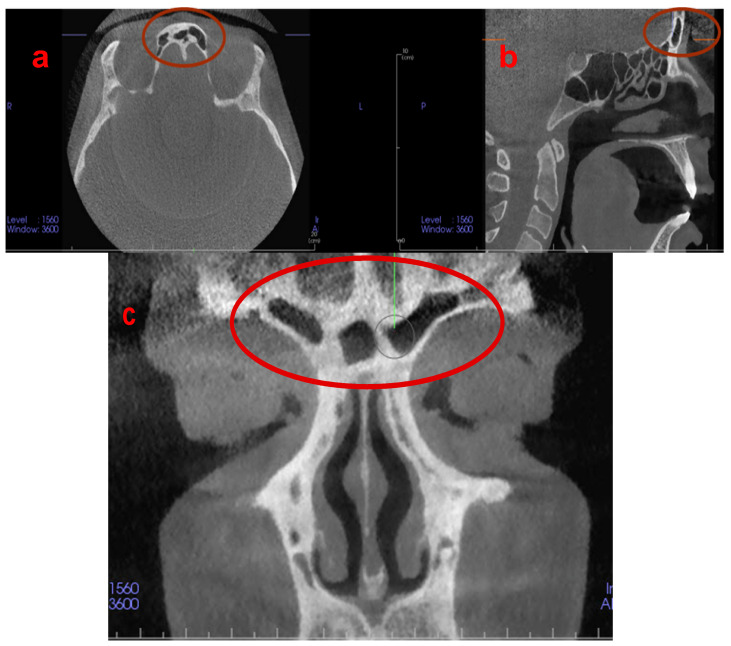
(**a**) Axial, (**b**) sagittal, and (**c**) coronal CBCT images demonstrating the unique and individualised architecture of the frontal sinuses.

In recent decades, the morphology of frontal sinuses (FS) has emerged as a crucial tool in forensic dentistry due to its dimensional stability in adulthood [8]. The FS are protected from injuries as an internal bony structure and even exhibit variations in monozygotic twins [9,10]. FS are bilateral air-filled cavities, and their initial development occurs at the age of 2 or 3 years. The FS can be clearly visualised radiographically at the age of 5 or 6 years, and their final shape is completed and fully developed at the age of 20 years [8,11,12] (Figure 1).

Cone beam computed tomography (CBCT) is a diagnostic imaging technique based on a series of cross-sectional images of a specific region (head–neck) with lower radiation than medical computed tomography (MDCT) [13,14]. CBCT’s application has increased since its commercial introduction in dentistry, and this prospect is vital for human identification, when it is technically feasible to exploit its full potential [15]. Among its advantages is the absence of overlapping structures, allowing detailed and clear imaging of each slice of the region of interest (ROI). This feature has been used repeatedly in the field of anthropology [16,17]. The uniqueness of frontal sinuses would significantly contribute to forensic science. Although the usefulness of the frontal sinuses for human identification has been the subject of many studies in the past, there is a need for a critical evaluation and systematic presentation of how the morphology of the frontal sinuses, as assessed by CBCT, can contribute to human identification.

The aim of this study is to investigate the existing literature and evaluate the contribution of frontal sinuses’ morphology (linear and volumetric measurements) to assist gender identification based on CBCT images.

## 2. Materials and Methods

### 2.1. Design

The present systematic review of the literature was conducted on the potential value of frontal sinuses in gender identification, evaluating data from cone beam computed tomography (CBCT) scans and their application in forensic science. This systematic review was performed in accordance with the PRISMA statement (Preferred Reporting Items for Systematic Reviews and Meta-Analyses) [18].

### 2.2. Eligibility Criteria

Inclusion and exclusion criteria were implemented.

#### 2.2.1. Types of Participants

Healthy individuals with free medical records were included. No age, gender, or ethnic group restrictions were applied.

#### 2.2.2. Types of Outcome Measures

The researchers designed data collection tables to record the most important information from each paper (sample size, ethnicity, number of males and females, mean age (range), planes of CBCT utilised, measurements conducted, software used).

#### 2.2.3. Study Design

Only full-text case reports and clinical trials on humans available in English were accepted. In vitro and technical notes were also accepted.

Experimental studies that satisfied the criteria of the systematic review were included. The follow-up period was not considered.

#### 2.2.4. Inclusion Criteria

The following inclusion criteria were implemented: full texts of case reports, technical notes, in vitro studies, experimental and clinical studies on humans available in the English language were included.

#### 2.2.5. Exclusion Criteria

Personal opinions, author debates, letters to the editor, author responses, books and/or book chapters, news group papers, abstracts, editors’ summaries, congress abstracts, summary papers, experimental studies on animals, non-English papers, and papers evaluating panoramic or conventional imaging techniques were all excluded. Papers combining CBCT with panoramic, medical computed tomography, or other dental imaging modalities were not considered.

### 2.3. Information Sources

The following electronic databases were comprehensively searched from January 2010 to June 2022: Scopus, PubMed, Web of Science, and Cochrane Library.

### 2.4. Search Strategy

**Figure 2:**
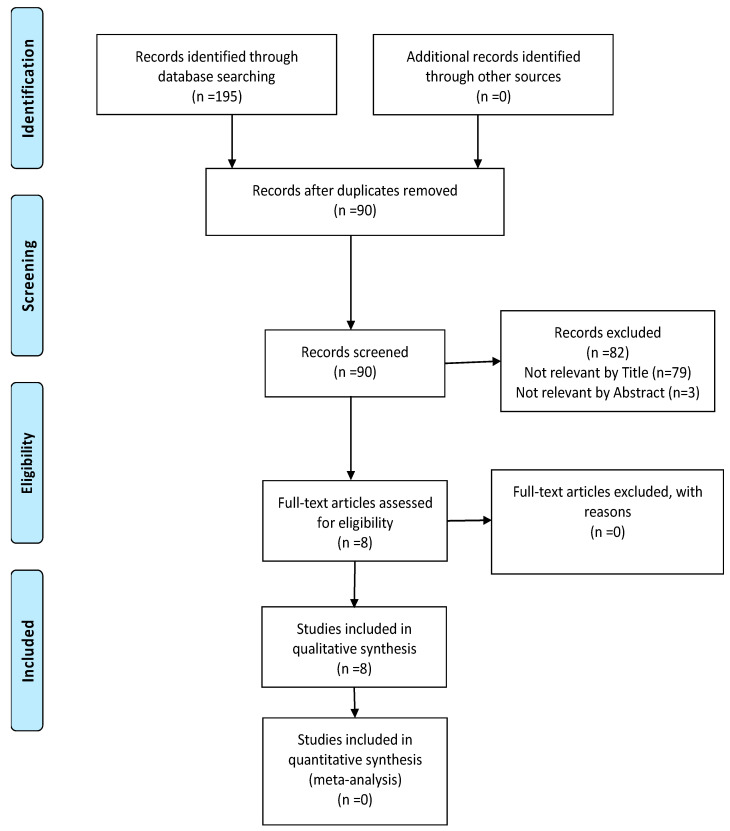
PRISMA flow diagram.

Two observers (A.M., N.C.) conducted the systematic search utilising suitable medical subject headlines (MeSH) and free text synonyms. The web-based literature research was confined to English-language papers published between January 2010 and June 2022. Figure 2 depicts the entire electronic search approach in detail (PRISMA inflow illustration). Search inquiries were created exercising the Boolean operators AND and OR, as well as the expressions “anterior sinuses”, “cone beam computed tomography”, “forensic identification”, and “gender”, as well as their conceivable combinations. Additionally, no intervention was made throughout the procedure, and the articles were acquired using rigorous selection and exclusion criteria.

### 2.5. Study Selection

Duplicates in studies collected from these databases were examined. The study selection process was divided into two phases. In the first phase, two reviewers (A.M., N.C.) thoroughly and independently examined the titles and abstracts retrieved from all electronic databases on the potential contribution of the morphology of frontal sinuses to gender identification based on cone beam computed tomography images. In the case of disagreement on which articles to screen with the full text, a consensus was achieved through discussion. If necessary, the final decision was made after consultation with a third reviewer (A.R.). In the second phase, full-text articles were assessed independently by two reviewers (A.M., N.C.) for inclusion. Any disagreement concerning full text inclusion was resolved with discussion and, if necessary, with consultation with the third reviewer (A.R.) until consensus was reached. Each rejected paper’s reasons for rejection were listed separately.

### 2.6. Data Collection and Data Items

The same two review authors (A.M., N.C.) collected the data independently in a customised and pre-defined data extraction table. Extracted data were compared, and in case of discrepancies, a consensus was reached through discussion and the re-examination of the studies. The data extraction form included the following items: general information (authors’ names, publication year, journal, ethnicity); most essential information from each article (planes of CBCT used, type of measurements performed, software used); and participants (sample size, number of males and females, age). Data extraction was carried out autonomously and in duplicate by the same two authors (A.M., N.C.) from all the articles that were ultimately included in the study. Any disputes that arose during data collection were resolved through discussion with the involvement of a third author.

### 2.7. Risk of Bias Assessment in Included Studies

The quality assessment of the included studies was carried out by two reviewers (A.M., N.C.) independently, using the risk of bias in non-randomized studies of interventions (ROBINS-I) tool for non-randomized trials [19]. The likelihood of bias in each study was independently assessed and confirmed by two of the authors. Then, the authors carefully discussed any differences to come to an agreement. The article was sent to a third author (A.R.) for the final evaluation on quality ratings if the two authors were unable to agree.

### 2.8. Effect Measures and Data Synthesis

The primary outcome of this systematic review was to investigate the contribution of the morphology of frontal sinuses to gender identification based on CBCT images. Variables extracted from each article were the following: author and year of publication, gender, mean age, sample size, planes of CBCT, software used, and outcome assessment tool (linear/volumetric measurements).

## 3. Results

### 3.1. Description of Studies

The results of the literature search, identification, inclusion, and exclusion of the articles are presented in the flow diagram according to the PRISMA statement (Figure 2). The electronic and manual search initially identified 195 relevant records, and 90 remained after a manual duplicate check. Eight articles were selected for a full review according to the inclusion and exclusion criteria after title and abstract screening.

The articles included were published between 2016 and 2022, with the highest number of publications on 2018. Of the eight articles identified, three reported data from an Indian population, two from a Brazilian population, and one from Saudi Arabian, Romanian, and Egyptian populations. In all articles, the male-to-female sample ratio was 1:1, except for the study by Sangavi et al. (2021), who did not report their sample composition [17,20,21,22,23,24,25,26].

The sample size ranged from 30 to 163 subjects. Of the eight articles identified, the majority of them assessed samples of more than 100 participants, except for Benghiac et al.’s (2017) and Motawei et al.’s (2016), who reported results of 30 and 53 participants, respectively. A total of 718 participants (359 males and 359 females) were retrieved from the included studies (Table 1) [17,20,21,22,23,24,25,26]. Since each article was analysed independently and there was heterogeneity between them, it was impossible to perform a meta-analysis.

Of the eight articles identified, three reported that measurements of the morphology of the frontal sinuses was a highly accurate method that correctly identified an individual’s gender [17,20,21], while the other five articles claimed that measuring specific volumetric and linear parameters of frontal sinuses can be utilised as a reliable method in the identification process [21,23,24,25,26].

Additionally, of the eight articles identified, two reported findings evaluating only volumetric measurements of the frontal sinuses [20,24], two reported findings calculating only linear measurements of the frontal sinuses [22,25], and four reported findings by combining both linear and volumetric measurements of the frontal sinuses (MS) [17,21,23,26].

Chatra et al. (2020) and Rao et al. (2022) [22,24] suggested a gender estimation equation that correctly identifies 64.1% and 63.1% of total cases, respectively.

Three-dimensional data were registered using diverse types of software programs, e.g., SOREDEX software [22], Planmeca Romexis software [23,24,25,26], InVesalius program [17], Anatomage In vivo 5.1 software [21], and ITK-SNAP software (version 2.1.4) [20].

**Table 1:** Data extraction from the included in the review studies.

Authors	Year of Publication	Article Title	Sample Size	Ethnicity	Journal	Gender	Mean Age	Planes of CBCT	Volume/Dimension	Software
Rao et al. [22]	2022	Evaluation of Frontal Sinus Index in Establishing Sex Dimorphism Using Three-Dimensional Cone Beam Computed Tomography in Northern Saudi Arabian Population	150	Saudi Arabian	Journal of Forensic Science and Medicine	74 M 76 F	32.5 years	Mid- Sagittal plane	Linear Measurements	Soredex
Sangavi et al. [23]	2021	Morphometric analysis of orbital aperture and frontal sinus using cone beam computed tomography as an aid in gender identification in forensic dentistry: a retrospective study	100	Indian	Journal of Punjab Academy of Forensic Medicine and Toxicology	Not mentioned	Not mentioned, range 18–65 yrs	Axial, Coronal Sagittal	Volume/Linear Measurements	Planmeca Romexis
Chatra et al. [24]	2020	Sexual dimorphism in frontal sinus volume: A CBCT comparative study	92	Indian	Journal of Indian Academy of Forensic Medicine	46 M 46 F	Not mentioned	3D reconstructions	Volume	Planmeca Romexis
Wanzeler et al. [20]	2019	Sex estimation using paranasal sinus discriminant analysis: a new approach via cone beam computerized tomography volume analysis	163	Brazilian	International Journal of Legal Medicine	80 M 83 F	Not mentioned	Axial, Coronal Sagittal, 3D reconstructions	Volume	ITK-SNAP,2.1.4 CS 3D Imaging 3.2.9
Choi et al. [17]	2018	The Frontal Sinus Cavity Exhibits Sexual Dimorphism in 3D Cone-beam CT Images And can be Used for Sex Determination	130	Brazilian	Journal of Forensic Sciences	65 M 65 F	Not mentioned, range 18–78 yrs	Axial, Coronal Sagittal, 3D reconstructions	Volume/Linear Measurements	In Vesalius program, ImageJ software
Denny et al. [25]	2018	Frontal Sinus as an aid in Gender Identification in Forensic Dentistry: A Retrospective Study using Cone Beam Computed Tomography	100	Indian	World Journal of Dentistry	50 M 50 F	Not mentioned, range 20–30 yrs	Axial, Coronal Sagittal	Linear Measurements	Not mentioned (Promax 3 Dmid Unit)
Benghiac et al. [26]	2017	CBCT assessment of the frontal sinus volume and anatomical variations for sex determination	30	Romanian	Romanian Journal of Legal Medicine	15 M 15 F	39.4 ± 14.8 yrs	Axial, Coronal Sagittal, 3D reconstructions	Volume/Linear Measurements	Romexis 4.4.0.
Motawei et al. [21]	2016	Assessment of frontal sinus dimensions using CBCT to Determine sexual dimorphism amongst Egyptian population	53	Egyptian	Journal of Forensic Radiology and Imaging	29 M 24 F	32.35 ± 10.82 yrs	Axial, Coronal	Volume/ Linear Measurements	Anatomage In vivo 5.1.

M: males, F: females.

### 3.2. Risk of Bias

The risk of bias is summarized for the eight studies that were implemented into the final assessment in Table 2 [17,20,21,22,23,24,25,26]. In terms of overall risk of bias, a moderate risk was assigned to the six of the eight articles. Just two of the eight articles were classified as low risk. Most of the included studies seemed to have a major methodological problem due to the lack of standard measurement protocol concerning the frontal sinuses, and the sample size was small.

## 4. Discussion

In the current review, an extended survey of the previous literature on frontal sinuses’ contribution to gender identification was attempted by evaluating CBCT records among four different electronic databases.

CBCT imaging technology provides clear and precise images of the head and neck region. Anatomical components are not superimposed in CBCT images. As a result, since the third dimension is available, this radiographic evaluation outperforms 2D radiographs. The primary drawback of CBCT is that the patient is exposed to a higher radiation dose. Hence, this radiographic examination should always be considered, and careful selection criteria must always be followed [13,14,15,16,17].

Choi et al. (2018) evaluated three-dimensional reconstructions obtained from 130 CBCT examinations [17]. The selected images were obtained with an iCAT Classic model apparatus (Imaging Sciences International, Hatfield, PA, USA). CBCT scans were selected according to the inclusion and exclusion criteria. The initial segmentation method was performed with the InVesalius program. Subsequently, images were generated in frontal, lateral, and basal views, and their pixel calculation was performed with a second program (ImageJ). Different aspects of each image were described by several parameters, such as area, perimeter, bounding rectangle, occupied area, ellipse fit, circularity, aspect ratio, roundness, solidity, and Feret’s diameter. Statistical analysis revealed a high level of accuracy (80%) in gender determination. The basal and frontal aspects of the three-dimensional model had a significant contribution to the final model. Additionally, the volume parameter was identified as a statistically significant variable in determining gender, and this high level of accuracy was valid for both males and females [17]. Nevertheless, the article has certain limitations. The authors did not report examiners’ experience, and cranial measurements analysis were not considered.

Wanzeler et al. (2019) aimed to assess paranasal sinuses’ volume (maxillary, frontal, and sphenoidal) based on a discriminant analysis to determine gender estimation [21]. One-hundred and sixty-three (163) cranial CBCT scans obtained using the I-Cat^®^ tomography apparatus (Imaging Sciences-Kavo, Hatfield, PA, USA) operating at 110 kV, 40 mA, and belonging to the archives of a dental clinic were analysed by two experienced maxillofacial radiologists. Each paranasal sinus’ region was delineated according to the anatomical borders. Images were evaluated using ITK-SNAP,2.1.4 software, appropriate for reliable measurements. This software produced a full complement of the delimited area. Axial, sagittal, and coronal planes were retrieved for optimal visualisation. After complete sinus segmentation, volume was displayed in cubic centimetres and all measurements were performed using the CS 3D Imaging 3.2.9 software. Statistical analysis revealed that the frontal sinuses’ measurements (volume) were performed with an overall gender estimation accuracy of 94.4%. When correlating their findings with foramen magnum measurements, the accuracy rate of gender identification increased to 100%. Their results are extraordinarily high and are not consistent with the studies of Uthman et al. (2010) and Michel et al. (2015), who reported that FS analysis could accurately determine an individual’s gender in 76.9% of cases, whereas the accuracy rate increased to 85.9% by combining other skull-specific measurements [11,27]. In the study by Michel et al. (2015), the accuracy of gender determination decreased to 72.5% [27].

In the retrospective study by Benghiac et al. (2017), volumetric measurements of FS were evaluated to establish a new technique for gender estimation [26]. The CBCT scans were performed using Planmeca ProMax 3D Mid (Planmeca, Helsinki, Finland), and the acquisition protocol was tailored to include the anatomical areas of interest that correspond to the FS. DICOM records of thirty adult Romanian patients (aged 18–65 years old) were analysed, according to inclusion criteria. CBCT images, obtained from the DICOM format, were transferred to a personal computer. Two independent and experienced examiners analysed the acquired images and used the “ellipse” feature of Romexis 4.4.0 (Planmeca, Helsinki, Finland) for volumetric measurements in coronal, sagittal, and axial reconstructions. They created and measured three different volumes of FS (V1, V2, V3) with similar prediction levels. The results of their research indicated a cut-off value for each volume (7200, 7221, and 7170 for V1, V2, and V3, respectively) regarding gender determination, which corresponded to an extremely high rate of sensibility and a satisfactory specificity rate. Values below the cutoff values were therefore associated with female prediction with a probability of more than 80%. The study is limited in various ways. The sample is characterised as extremely small, and the accuracy prediction for males is only approaching 67%. A larger sample would be preferable to provide more reliable conclusions. The authors suggested these volumetric measurements of FS as a valuable resource for gender determination.

Chatra et al. (2020) aimed to assess sexual dimorphism of FS, and reported a significant volume difference between males and females [24]. In their retrospective radiographic study, one hundred and forty CBCT scans were analysed retrieving data from a sample size of ninety-two subjects (46 females and 46 males). Individuals who underwent surgery on the frontal sinuses, and CBCT images presenting pathologies, developmental disorders and/or facial deformities were excluded. The Romexis software of the Planmeca ProMax 3D mid CBCT machine was used to study the frontal sinus. The volume was measured directly by two experienced examiners using a special tool (ellipse feature) provided by the CBCT Romexis software. This specific software recognized septa and separated FS areas, providing more accurate measurements. The authors proposed an equation for gender prediction (Sex = 0.936 − (0.116) × volume). Thus, values less than the cut-off value related to male prediction. Chatra et al. (2020) considered their technique as a reliable method for sexual dimorphism in the Indian population [24]. The method’s accuracy is limited because their results did not demonstrate high accuracy prediction in males (54.3%), and there was no statistically significant difference in mean total volume between genders.

According to the study by Sangavi et al. (2021), specific linear parameters such as “left FS length” and “left FS height” demonstrated a statistically significant difference between genders [23]. Their evaluation included both morphological and volumetric parameters. For morphological parameters, the examiners performed axial and coronal reconstructions, while for dimensional, they used the “ellipse” feature of Planmeca Romexis software in coronal, sagittal, and axial reconstructions. They also evaluated measurements of the orbit (maximum height, width, index, interorbital distance), which were estimated in coronal planes. For their study, they analysed one hundred CBCT images from subjects aged 18 to 65 years old. Despite significant variations in orbital and FS dimensions, the authors considered their method as reliable for gender assessment. In contrast, Denny et al. (2018) and Rao et al. (2022) measured linear parameters (in mm) of the FS region (maximum width–height and height, width at maximum dimensions and their ratio is considered as a frontal sinus index, respectively) [22,25].

In the study by Denny et al. (2018), two linear parameters of FS (width and height in maximum dimension) were calculated in the sagittal, axial, and coronal planes of CBCT images [25]. The examinations were carried out using the Promax 3DMid (Planmeca Oy., Helsinki, Finland) CBCT unit. The radiographs were taken at 90 kVp, 8 mA, and an exposure time of 27 s. A slice thickness of 0.400 mm was used to assess the sections. The exposure parameters according to the standard default values were based on the FOV. This retrospective study was conducted by evaluating 100 cone beam computed tomography (CBCT) images (50 males and 50 females, age range 20 to 30 years old). Measurements of the morphology of the frontal sinuses indicated that the measurements in males were larger than those in females, and also were larger on the left side than on the right. The reliability of the results was limited in several ways. All the included measurements in both right and left frontal sinuses on the axial, sagittal, and coronal sections were not statistically significant. Although these measurements were significantly higher in the male subpopulation, they were not statistically significantly different, and cannot be considered as a reliable indicator of sexual dimorphism.

Rao et al. (2022) assessed the potential correlation between linear measurements of FS and gender estimation using cone beam computed tomography (CBCT) images in the north Saudi Arabian population [22]. Three-dimensional CBCT scans of one hundred and fifty adult participants (74 men and 76 women, mean age 32.5 years) were retrospectively analysed. All the scans were selected from the database of head-and-neck 3D-CBCT explorations, which had a similar standardised protocol of acquisition and were attained using Cranex (SOREDEX, Tunusula, Finland) with an imaging protocol or field of view measuring 16 cm diameter × 13 cm height, 0.25 mm slice thickness, and a scanning time of 20 s. Linear parameters (width and height) were measured in the mid-sagittal plane with the accompanying software SOREDEX, and their ratio was considered as a frontal sinus index. According to their statistical analysis (discriminant function analysis and binary logistic regression), the variables “height” and “length” were not statistically significant different between males and females. The mean width of the frontal sinus in men (13.39 ± 3.6 mm) was slightly higher than that in females (12.06 ± 3 mm). The authors recommended a gender estimation equation (n = −9.96 + (−0.378 × frontal sinus height) + (0.979 × frontal sinus width) + (3.58 × ratio) with 63.1% correct classification. The critical cut-off value was determined to be 0.321 (equal to or greater than 0.321, gender prediction indicated male, while for females it was less than 0.321). The main limitations of their study were that they proposed the frontal sinus index parameter as a contributing factor to gender estimation without it being statistically significant. In addition, they did not report the accuracy rate for each gender separately, but overall.

In the study by Motawei et al. (2016), statistically significant differences were identified between males and females in measurements of frontal sinus height (HFS), width of the FS (WFS), and length of FS (LFS) [21]. The device used in this study was an iCAT Next Generation, Imaging Science International, Hatfield, PA, USA. The imaging protocol used was 16 cm diameter × 13 cm height (field of view), 0.25 mm voxel size, with a scanning time of 14.7 s. For their study, they selected fifty-three adult subjects (29 males and 24 females aged between 20 and 58 years old), according to inclusion criteria. Patients with pathological features, nasal sinus disease, and mucosal thickening were excluded. Four examiners measured six linear parameters in three-dimensional reconstructions of samples’ CBCT images with Anatomage In vivo 5.1 software in the coronal, and axial planes. They also examined presence or absence of FS, scalloping, and presence or absence of sinus septum. The authors reported a simple linear regression model in which all variables were statistically significant, except the variable “distance between the highest points of the sinuses”. A potential limitation of the proposed method was that no statistically significant difference was observed in FS descriptive data (sinus septum, scalloping) between sexes.

The dimensional uniqueness of FS (volumetric and morphological features) obtained from CBCT images was assessed through extended literature review. The results of this systematic research indicated significant variation in frontal sinuses. In our study, all authors agreed that FS measurements contributed to gender determination. In particular, FS anatomy is a valuable resource for sex determination when comparing post-mortem and antemortem images. These results are in agreement with the findings of previous studies [7,13,28,29,30,31,32,33,34,35,36,37,38,39]. Volumetric estimation differs significantly between genders, and can be considered as a reliable method for gender identification. These results agree with the results of previous studies [11,13,33,34,37,40,41,42].

Studies conducted by Yoshino et al., Verma et al., and Goyal et al. reported statistically insignificant differences between the sex dimorphism and frontal sinus measurements [8,12,43].

The results of the current review are not consistent with previous results reported by Zhao et al. (2021) [44]. Their findings revealed no statistically significant differences in morphological measurements between genders [44].

There are a few limitations considering the results of this systematic review. The dimensions of FS change over time and are affected by trauma, surgery, and disease. CBCT is a new diagnostic imaging technique based on a series of cross-sectional images and provides numerous advantages. However, frontal sinuses are not always located within the region of interest (ROI). Another potential limitation is that there were neither accurate protocols for specific measurements nor known error rates of each procedure. Post-mortem imaging is not a standard condition in all countries.

## 5. Conclusions

According to the current review, the dimensions of frontal sinuses demonstrate anatomical differences between the genders. In particular, the volume and the height of the frontal sinuses is significantly greater in males than in females. Consequently, these parameters can be used as a complementary method to identify the gender of unknown corpses.

Volumetric and linear measurements of the frontal sinuses can be used as a supplement to determine gender. Due to the precision and accuracy of the linear and volumetric measurements that it provides in anatomical structures such as frontal sinuses, cone beam computed tomography (CBCT) is a recommended diagnostic tool for gender estimation. A larger sample size and common measurement protocols are needed to verify its usefulness.

## Figures and Tables

**Table 2:** Risk of bias of included non-randomized studies according to ROBINS-I tool [19].

				Bias Due to/in…				
Title 1	Confounding	Selection of Participants for the Study	Classification of Interventions	Deviations from Intended Interventions	Missing Data	Measurement of Outcomes	Selection of the Reported Result	Overall
Choi et al. 2018 [17]	Low	Moderate	Low	Low	Low	Low	Low	Moderate
Denny et al. 2018 [25]	Low	Low	Low	Low	Low	Low	Low	Low
Motawei et al. 2016 [21]	Low	Moderate	Low	Low	Low	Low	Moderate	Moderate
Sangavi et al. 2022 [23]	Low	Moderate	Low	Moderate	Low	Low	Low	Moderate
Wanzeler et al. 2019 [20]	Low	Moderate	Low	Moderate	Moderate	Low	Low	Moderate
Chatra et al. 2020 [24]	Low	Low	Low	Low	Low	Low	Low	Low
Rao et al. 2022 [22]	Low	Moderate	Low	Moderate	Low	Moderate	Moderate	Moderate
Benghiac et al. 2017 [26]	Low	Moderate	Low	Moderate	Low	Moderate	Moderate	Moderate

## Data Availability

Data are available on request from the corresponding author.
